# Superstructures with cyclodextrins: Chemistry and applications IV

**DOI:** 10.3762/bjoc.13.215

**Published:** 2017-10-18

**Authors:** Gerhard Wenz

**Affiliations:** 1 Saarland University, Organic Macromolecular Chemistry, Campus C4 2, 66123 Saarbrücken, Germany

**Keywords:** cyclodextrins, superstructures

Superstructures are generally formed from molecules through non-covalent interactions, which can be either attractive or repulsive. The first examples of superstructures formed by attractive intermolecular forces were complexes of crown ethers [1], cryptands [2] and spherands [3], which were recognized by the 1987 Nobel Prize in Chemistry. In addition, inclusion compounds of cyclodextrins (CDs), cyclic oligomers of glucose, belong to this category and have been known since the pioneering work of Friedrich Cramer in 1951 [4–5]. Superstructures can also be held together by repulsive forces, so-called mechanical bonds [6], as exemplified in catenanes, rotaxanes, and knots [7]. Because of their restricted mobility, rotaxanes are well-suited for the design of molecular machines, which were the subject of the 2016 Nobel Prize in Chemistry [8–9]. Since superstructures are accessible through rational design, supramolecular chemistry had a great influence on organic and macromolecular chemistry as well as pharmacology and materials sciences. CDs in particular became the most important building blocks for superstructures because they are the only hosts that are non-toxic and available on an industrial scale. Recent developments in this exciting field are highlighted in the following.

α-CD forms one-dimensional superstructures with gold salts, such as KAuBr_4_, which readily crystallize as fine needles [10]. Based on this finding, a new and environmentally friendly recovery process for gold from excavation material is under development.

Significant progress was achieved in the functionalization of CDs through the regioselective introduction of one, two, three or more equal or different substituents starting from the corresponding perbenzyl or permethyl derivatives [11–12]. In this way, hexaphyrine was attached threefold to α-CD to construct superstructures with switchable aromaticity [13]. Furthermore, buckminsterfullerene C_60_ was covalently linked to two γ-CD rings [14]. This conjugate is highly soluble in water because it forms a sandwich-like self-complex, which makes it particularly useful as a sensitizer for singlet oxygen ^1^O_2_ generation [14].

Amphiphilic CD derivatives with oligoethylene oxide side chains form nanoparticles with the sensitizer Zn-phthalocyanine and the antineoplastic drug docetaxel. These materials might be applicable for dual cancer therapy [15]. Another self-assembled CD nanocarrier for the photoreactive dye squaraine that is useful for photodynamic therapy is described in this Thematic Series [16]. The group of Ravoo also conjugated arylazopyrazoles to amphiphilic cyclodextrin derivatives that form vesicles triggered by light [17]. A star-shaped polycationic CD derivative with many breakable, intrinsic, disulfide linkages forms nanoparticles with messenger RNA and drugs and is particularly useful for transport into HeLa cells [18].

Due to their hydrophilicity, CD-derived superstructures have become more and more interesting for the construction of biomaterials. In particular, interactions between cellular components and CDs might lead to “greener medical applications” as summarized in this Thematic Series in the article by Leclercq [19]. For example, β-CD was immobilized onto a silicon substrate and incubated with various bacteria. These bacteria could be captured nearly quantitatively at the surface and released again by competitive binding with free methylated β-CD [20]. The proteins insulin and lysozyme were also conjugated to the guest adamantane. The complexation of these conjugates by pegylated β-CD gives rise to superstructures which provide slow release and maintain full biological activity [21].

Significant progress was also achieved in the field of CD rotaxanes. A [3]-rotaxane was assembled from two monosubstituted α-CD rings, α,ω-dodecamethylene diazide axis, and two bulky alkyne stoppers through a copper-catalyzed [2 + 3] cycloaddition in one step. Because of the attached gadolinium complexes, this rotaxane showed a high NMR relaxivity, making it suitable as a probe for magnetic resonance imaging (MRI) [22]. Well-defined fluorescent polyrotaxanes with alternating, threaded cucurbit[6]uril and CD rings were assembled by Fraser Stoddart’s group via an alkyne–azide click reaction exploiting supramolecular catalysis [23–24]. The concurrent radical copolymerization of 1,3-dienes and bulky stoppers in the presence of CDs (so-called *rotaxa*-polymerization) was further applied for the syntheses of water-soluble polyisoprene polyrotaxanes [25]. Furthermore, ABA-type block-copolyrotaxanes could be synthesized using this polymerization technique controlled by RAFT chain transfer agents [26].

The exciting world of CD polyrotaxanes, including self-healing materials [27], was highlighted at a special session “Smart Polymers and Materials from Cyclodextrins: Novel Designs and Functions” at the 253rd ACS National Meeting in San Francisco in spring 2017. This Thematic Series in the *Beilstein Journal of Organic Chemistry* also provides further insights into the synthesis and properties of CD superstructures, covering many aspects such as catalysis, molecular recognition, colloids, polyrotaxanes, drug delivery and more.

Gerhard Wenz

Saarbrücken, September 2017

## References

[1] Pedersen C J, Frensdorff H K (1972). Angew Chem.

[2] Lehn J-M (1978). Acc Chem Res.

[3] Cram D J (1988). Angew Chem.

[4] Cramer F (1951). Chem Ber.

[5] Cramer F, Hettler H (1967). Naturwissenschaften.

[6] Bruns C J, Stoddart J F (2016). The Nature of the Mechanical Bond: From Molecules to Machines.

[7] Sauvage J, Dietrich-Buchecker C (1999). Molecular Catenanes, Rotaxanes and Knots.

[8] Stoddart J F (2017). Angew Chem, Int Ed.

[9] Sauvage J-P (2017). Angew Chem, Int Ed.

[10] Liu Z, Samanta A, Lei J, Sun J, Wang Y, Stoddart J F (2016). J Am Chem Soc.

[11] Guieu S, Sollogoub M (2008). J Org Chem.

[12] Wang B, Zaborova E, Guieu S, Petrillo M, Guitet M, Blériot Y, Ménand M, Zhang Y, Sollogoub M (2014). Nat Commun.

[13] Ménand M, Sollogoub M, Boitrel B, Le Gac S (2016). Angew Chem, Int Ed.

[14] Zhu X, Quaranta A, Bensasson R V, Sollogoub M, Zhang Y (2017). Chem – Eur J.

[15] Conte C, Scala A, Siracusano G, Sortino G, Pennisi R, Piperno A, Miro A, Ungaro F, Sciortino M T, Quaglia F (2016). Colloids Surf, B.

[16] Kauscher U, Ravoo B J (2016). Beilstein J Org Chem.

[17] Stricker L, Fritz E-C, Peterlechner M, Doltsinis N L, Ravoo B J (2016). J Am Chem Soc.

[18] Yang W, Yu C, Wu C, Yao S Q, Wu S (2017). Polym Chem.

[19] Leclercq L (2016). Beilstein J Org Chem.

[20] Perez-Anes A, Szarpak-Jankowska A, Jary D, Auzély-Velty R (2017). ACS Appl Mater Interfaces.

[21] Hirotsu T, Higashi T, Motoyama K, Arima H (2017). Carbohydr Polym.

[22] Fredy J W, Scelle J, Ramniceanu G, Doan B-T, Bonnet C S, Tóth É, Ménand M, Sollogoub M, Vives G, Hasenknopf B (2017). Org Lett.

[23] Hou X, Ke C, Zhou Y, Xie Z, Alngadh A, Keane D T, Nassar M S, Botros Y Y, Mirkin C A, Stoddart J F (2016). Chem – Eur J.

[24] Hou X, Ke C, Stoddart J F (2016). Chem Soc Rev.

[25] Hilschmann J, Kali G, Wenz G (2017). Macromolecules.

[26] Hilschmann J, Wenz G, Kali G (2017). Beilstein J Org Chem.

[27] Kato K, Mizusawa T, Yokoyama H, Ito K (2017). J Phys Chem C.

